# The effect of catheter-based sham renal denervation in hypertension: systematic review and meta-analysis

**DOI:** 10.1186/s12872-023-03269-w

**Published:** 2023-05-12

**Authors:** Adriana Fernandes, Cláudio David, Fausto J Pinto, João Costa, Joaquim J Ferreira, Daniel Caldeira

**Affiliations:** 1 Faculdade de Medicina, Universidade de Lisboa, Lisbon, Portugal; 2 Laboratory of Clinical Pharmacology and Therapeutics, Faculdade de Medicina, Universidade de Lisboa, Lisbon, Portugal; 3 Instituto de Medicina Molecular, Faculdade de Medicina, Universidade de Lisboa, Lisbon, Portugal; 4 Faculdade de Medicina, Centro Cardiovascular da Universidade de Lisboa (CCUL@RISE), Universidade de Lisboa, Lisbon, Portugal; 5 Cardiology Department, Hospital Santa Maria, Centro Hospitalar Univesitário Lisboa Norte (CHULN), Centro Académico de Medicina de Lisboa (CAML), Lisbon, Portugal; 6 CNS – Campus Neurológico Sénior, Torres Vedras, Portugal; 7 CEMBE (Centro de Estudos de Medicina Baseada na Evidência, Faculdade de Medicina da Universidade de Lisboa, Lisboa, Portugal

**Keywords:** Hypertension, Placebo, Renal denervation, Renal sympathetic denervation, Sham

## Abstract

**Background:**

Renal denervation (RDN) has emerged in recent years as a possible treatment for hypertension. The first sham-controlled trial showed a small magnitude and non-significant in the blood pressure (BP) lowering effect, also due to a substantial decrease of BP in sham arm. Considering this, we aimed to quantify the magnitude of BP decrease within the sham arm of Randomized Controlled Trials (RCT) with RDN in patients with hypertension.

**Methods:**

Electronic databases were searched since inception until January 2022 for randomized sham-controlled trials which assessed the efficacy in lowering BP of the sham intervention for catheter-based RDN in adult patients with hypertension. The outcomes were change in ambulatory/office systolic and diastolic BP.

**Results:**

A total of 9 RCT were included in the analysis enrolling a total of 674 patients. Sham intervention showed a decrease in all evaluated outcomes. Office systolic BP had a reduction of -5.52 mmHg [95%CI -7.91, -3.13] and office diastolic BP of -2.13 mmHg [95%CI -3.08, -1.17]. Sham procedure for RDN also showed a reduction of -3.41 mmHg [95%CI -5.08, -1.75] in ambulatory systolic BP and − 2.44 mmHg [95%CI -3.31, -1.57] in ambulatory diastolic BP.

**Conclusion:**

Despite recent data indicating that RDN might be an effective treatment for patients with resistant hypertension when compared to a sham intervention, our results indicate that the sham intervention for RDN also has a significant effect on lowering Office and Ambulatory (24-h) Blood Pressure in adult patients with hypertension. This highlights that BP itself might be sensitive to placebo-like effect and also brings further difficulties in establishing the BP lowering efficacy of invasive interventions due to the magnitude of the sham effect.

**Supplementary Information:**

The online version contains supplementary material available at 10.1186/s12872-023-03269-w.

## Introduction

Arterial hypertension remains one of the major preventable causes of cardiovascular disease and all-cause death globally, affecting over one billion people worldwide. Even in high-income countries, blood pressure (BP) control rates are low and multiple drug therapies are often needed, and in some cases insufficient, to achieve BP control [1, 2].

A number of different pathophysiological mechanisms are involved in the development of hypertension, amongst them, the activation of the sympathetic nervous system (SNS).

Renal SNS innervates the three major neuroeffectors in the kidney, leading to (1) increased renin secretion by juxtaglomerular granular cells via stimulation of β-1 adrenoceptors, (2) increased renal tubular sodium reabsorption and decreased urinary sodium excretion by renal tubular epithelial cells through stimulation of α-1B adrenoceptors and (3) reduced renal blood flow by stimulation of α-1 A-adrenoceptors on the renal vasculature [3, 4].

The established theory is that both renal sympathetic efferent and afferent nerves, which lie within and immediately adjacent to the wall of the renal artery, are crucial for initiation and maintenance of multiple patterns of hypertension mediated through noradrenaline, the primary neurotransmitter in efferent renal nerves, and substance P and calcitonin gene-related peptide as primary sensory neurotransmitters in afferent nerves. The SNS also plays an important role on the crosstalk between the kidney and the central nervous system (CNS) [3, 5].

In order to target these neurogenic mechanisms, many antihypertensive interventions have emerged either pharmacological, surgical or catheter-based. In this light, catheter renal denervation (RDN) was developed as a potential therapeutic option for patients with hypertension, by decreasing renal efferent sympathetic activation and interrupting or at least attenuating the afferent sensory signals to the CNS [3, 6, 7].

Up to this date the available data regarding the effectiveness of RDN in lowering blood pressure seems to be conflicting, especially when the intervention is compared with a sham control. While the first (non-blinded) clinical trials evaluating catheter-based RDN in patients with resistant hypertension showed promising results with decreases in systolic blood pressure (SBP) around 30 mmHg and 12–17 mmHg in diastolic blood pressure (DBP) [8, 9], when the intervention was compared with a sham procedure the results had a small magnitude and were not significant [10]. Sham procedures have an analogous purpose to placebo for drugs, by mimicking the actual therapeutic intervention, they are intended to neutralize biases such as the placebo effect [11]. This was one of the major proofs for the need of RCTs with sham-control particularly in arterial hypertension.

Although the SYMPLICITY HTN-3 trial failed to demonstrate a significant difference in BP reduction, the lack of difference was explained not by the absence of a decrease in BP after RDN but because the sham arm also showed a significant decrease in BP. These apparent results brought to light a possible effect induced by the sham intervention in lowering BP. Research in this area has also showed that the effect of placebo tends to be larger when the intervention in more invasive (as compared with pill placebo) [12]. Considering this, we found it essential to quantify the actual BP lowering effect of the sham intervention for RDN.

Therefore, we performed a systematic review to quantify the magnitude of blood pressure decrease within the sham arm of sham-controlled randomized clinical trials with catheter-based RDN in patients with hypertension.

## Methods

This systematic review was performed according to the Preferred Reporting Items for Systematic Reviews and Meta Analysis (PRISMA) guidelines for reporting systematic reviews evaluating health care interventions [13]. The protocol was registered in PROSPERO with the following record PROSPERO 2021 CRD42021244304.

### Eligibility criteria

We searched for published, randomized sham-controlled clinical trials, which assessed the efficacy of the sham intervention for catheter-based RDN in adult patients with resistant hypertension or other conditions suitable for renal denervation.

Given the different criteria used to define hypertension or elevated blood pressure, we accepted the definition used by the authors of each study. We also did not impose any restriction on previous or ongoing pharmacological treatment criteria throughout the trials.

Articles evaluating observational studies such as cohort, case-control studies, case-series, case reports and abstracts from conferences/congresses were excluded.

### Information sources and search strategy

We searched the electronic databases MEDLINE, Cochrane Central Register of Controlled Trials (CENTRAL) and Web of Science, from inception through January 2022 for eligible randomized sham-controlled trials. Complete search strategy can be found on Additional File Table 1.

Reference lists of articles and reviews were also analyzed to identify additional eligible studies. We did not apply restrictions in publication language.

### Study selection and data extraction process

Two reviewers screened the titles and abstracts of all trials retrieved in the electronic search, using the Rayyan review tool. In a second phase, the reports that met the criteria or were unclear were assessed through full text. Disagreements were solved by consensus between the 2 parties. The reasons for the exclusion of articles were recorded in both phases.

The data from the individual studies identified for inclusion was introduced into a pre-piloted form. This information included: authors and year of publication; study design; population details (age, gender, race, smokers, BMI, comorbidities – chronic kidney disease, coronary artery disease, peripheric artery disease, diabetes mellitus 2, obstructive sleep apnea, hyperlipidemia); outcomes of interest for this review; follow-up duration and mean number of antihypertensive agents. All data was retrieved from journal articles, trial protocols, Additional File, and clinical trial registries records.

### Risk of bias assessment

Two independent reviewers assessed the risk of bias in the included studies using the Cochrane Risk of Bias Tool for randomized trials (RoB 2) [14], based on 5 domains: randomization process, deviations from intended interventions, missing outcome data, measurement of the outcome and selection of the reported result. Each domain was classified as low risk, some concerns or high risk of bias.

Publication bias was also assessed through a funnel plot for all primary outcomes.

### Data synthesis and statistical analysis

Statistical analysis was performed with Review Manager 5.3 (The Nordic Cochrane Centre, The Cochrane Collaboration) and STATA 17. These programs were also used to derive forest plot showing the results of individual studies and pooled analysis.

The meta-analysis reported the pooled Mean Difference (MD) of blood pressure and 95% Confidence Interval (95% CI) evaluated through a random effects model due to the expected heterogeneity in the patients and devices. Statistical heterogeneity was assessed through the I [2] metric which measures the percentage of variation related to inter-study heterogeneity rather than random [15].

Jackknife sensitivity analysis was performed to assess the impact of each individual study in the pooled estimates. We performed subgroup analysis according to the risk of bias of included studies and according to whether trials were of first or second generation. Random-effects meta-regression was performed according to age, percentage of male patients, percentage of diabetic patients and number of anti-hypertensive drugs.

Small study effects/publication bias was assessed through the inspection of funnel plot and also by Egger test. In case a small-study effects was suspected by visual inspection of the funnel plot or Egger test results, we planned to follow the trim-and-fill method to assess publication/small-study effects bias in meta-analysis [16].

### Confidence in pooled data (grading of evidence)

The Grading of Recommendation, Assessment, Development, and Evaluation (GRADE) [17] criteria was used as the method for assessing the certainty in estimate of effect in the pooled evidence. Two reviewers evaluated risk of bias, inconsistency, indirectness, imprecision, and publication bias for all reported outcomes.

**Fig. 1:**
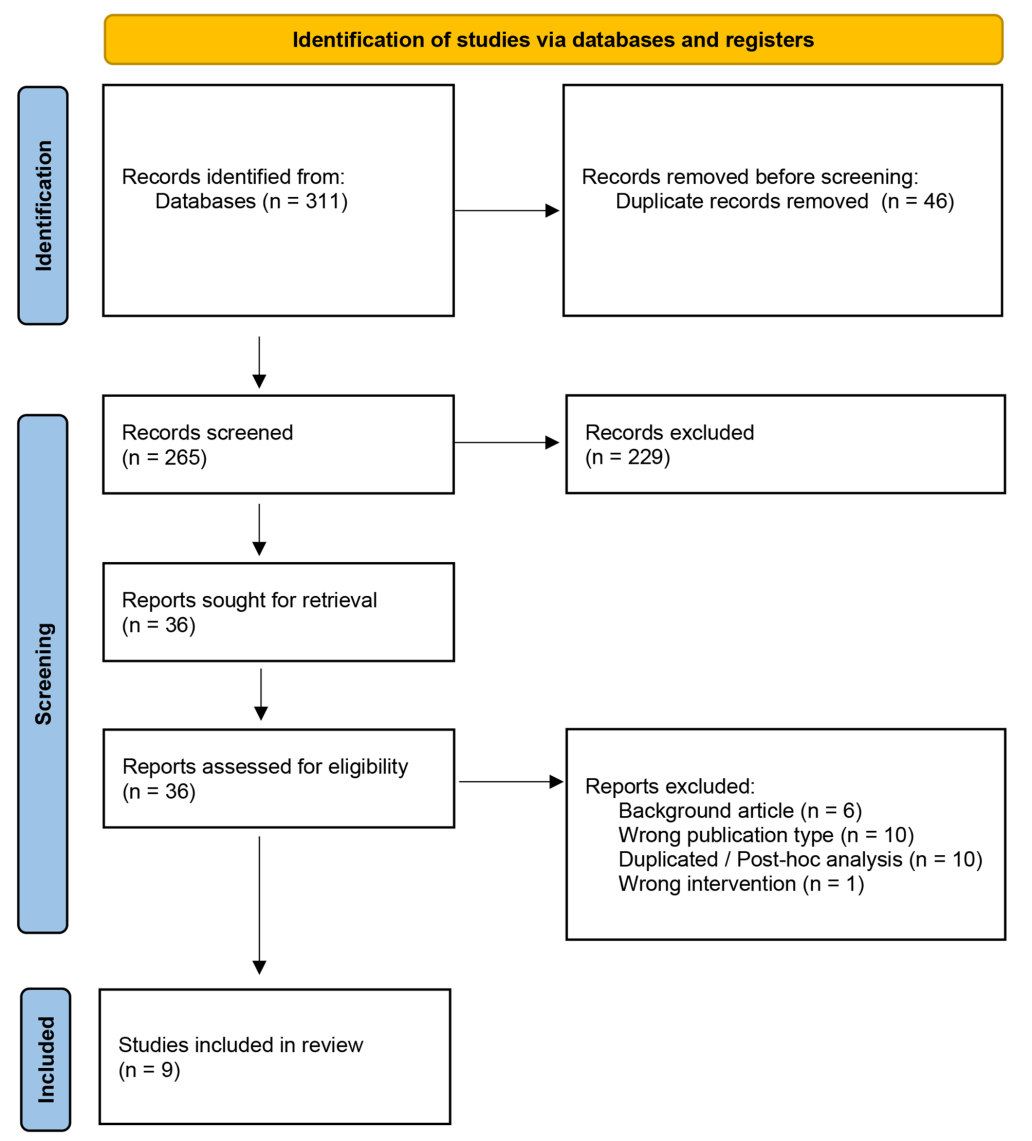
Flow diagram of study selection process

## Results

### Study selection

A total of 265 studies were retrieved through an electronic search of 3 databases (MEDLINE, Cochrane Central Register of Controlled Trials (CENTRAL) and Web of Science), of which 229 were excluded after reviewing titles and abstracts. The reasons for exclusion at this point were: wrong publication type (n = 93); animal studies (n = 43); wrong intervention (n = 24); duplicated / post-hoc analysis (n = 21); wrong outcome (n = 19); background article (n = 18); wrong study design (n = 5); unblinded follow-up of RCT (n = 5); interim analysis (n = 1).

After this phase, 36 studies were analyzed by full text of which 27 were excluded. Reasons for exclusion at this phase were: background article (n = 6); wrong publication type (n = 10); duplicated / post-hoc analysis (n = 10); wrong intervention (n = 1). The last one refers to a RCT where the RDN was achieved through an externally delivered focused ultrasound instead of a catheter-based device [18].

Nine RCTs met all the inclusion criteria and were eligible to be part of this systematic review. [10, 19–26] Schematic display of study selection process is depicted in Fig. 1.

### Study characteristics

Study characteristics are detailed in Table 1. Baseline demographics and main characteristics of populations are described in Table 2. Blood pressure reduction in RDN and control groups are described in Table 3.

All included studies were randomized sham-controlled trials, published from 2014 to 2021. A total of 674 patients were included in the sham arm of the trials. Eight of the 9 included trials used a Renal Angiography as the sham intervention, while one used a Radiograph Scan [20]. There were 5 international studies [21–23, 25, 26], 2 from USA [10, 24], 1 from Germany [19] and 1 from Denmark [20]. Follow-up duration ranged from 8 weeks to 6 months. Three of the RCTs were first-generation trials [10, 19, 20], while the other 6 comprised of second generation trials. The main differences between both generations consisted on second-generation RDN trials including procedures performed by highly experienced operators, employing more advanced radiofrequency ablation techniques and having stricter inclusion criteria of patients for the run-in phase and improved analysis of medication adherence [27].

From the 9 RCTs, a total of 674 patients underwent a sham intervention for RDN and 637 had available data for analysis (thirty-seven were lost because of declining follow up, missing or incorrect BP data and meeting trial’s escape criteria).

In the included trials mean patient age ranged from 52.6 to 58.2 years, between 30% and 100% of patients were white and from 54 to 81% were male. Regarding population comorbidities, from 11 to 26% were smokers, from 2 to 47% had coronary artery disease, from 6 to 31.6% had obstructive sleep apnea, from 5 to 40.9% had diabetes mellitus 2 and from 0 to 6% had peripheral artery disease. Mean body mass index (BMI) ranged from 28.4 to 33.9 kg/m^2^. Regarding Chronic Kidney Disease (CKD) 5 RCTs reported from 4 to 26.9% of patients with an estimated Glomerular Filtration Rate (eGFR) < 60 ml/min/1.73 m [2] and 2 RCTs reporting mean eGFR ranging from 84 to 86.2 ml/min/1.73 m [2]. However, eGFR was an exclusion criteria in all the RCTs (ranging from < 30 to < 45 ml/min/1.73m^2^).

Three RCTs were designed “Off Med”, meaning that patients had zero antihypertensive medication at the time of randomization [22–24]. Mean number of antihypertensive drugs was reported in four RCTs which ranged from 2.3 to 5.2. Two RCTs did not report the mean number of antihypertensive drugs having reported instead the percentage of patients taking each class of antihypertensive drugs [25, 26]. In most studies change in medication was not allowed during follow-up, except when patients met a predefined BP values considered to be too high, named “escape criteria”. In this situation previously established medication was administered for safety reasons. However, medication was allowed to be changed in one trial if patients asked for it, or if harmful changes in blood pressure, clinical appearances or biochemistry markers arose [20]. Three of the RCTs had medication adherence monitored through urine and blood testing [21, 23, 25].

Enrolment criteria differed in all trials, with the majority of studies including patients with resistant hypertension and high BP, although one study included patients with resistant hypertension but only slightly elevated BP (Ambulatory Systolic BP 135–149 mmHg and Ambulatory Diastolic BP 90–94 mmHg) [19].

Blinding of patients for their allocated group was not assessed in 2 trials and it was assessed in the other 7, all of them showing a James Blinding Index [28] superior to 0,5 which suggests significant blinding at discharge and at maximum follow-up. Although all of them reported successful blinding, two studies had a confidence interval at maximum follow up that intercepted 0.5, therefore with possible ineffective blinding [23, 24]. Only 2 trials [22, 25] have also assessed Bang’s Blinding Index [29], which showed an unsuccessful blinding in 3 out of 4 measurements in the intervention groups (> 0,2) and successful blinding in both sham groups (< 0.2), as described in Additional File Table 2.

The changes of blood pressure within each sham arm of the included randomized controlled trials are depicted in Table 3.

### Risk of bias in studies

Regarding the risk of bias assessment, four trials were considered as low risk of bias across all assessed domains.

Five studies had some concerns regarding measurement of the outcome since the trial funder was in charge of data collection and management [10, 21–23, 25].

The trial SPYRAL HTN-OFF MED Pivotal [23] was considered to have a high risk of bias, due to missing outcome data that may be dependent on its true value. Although it is stated in the Appendix of the trial that “multiple imputation sensitivity analysis was performed for the primary and secondary efficacy endpoints. Subjects with missing 3-month data had their values imputed by a regression model using baseline SBP, treatment group, age, gender, and BMI as covariates. One hundred imputed datasets were generated, and a pooled estimate of the treatment effect was calculated using Rubin’s Rule”, data for the outcome of change in 24 h BP was only available for 130 out of 165 patients (81,2%) and only these patients were included in the intention-to-treat (iTT) analysis in the sham group. Similarly, only 137 out of 166 patients (82,5%) were included in the iTT analysis in the renal denervation group.

**Fig. 2:**
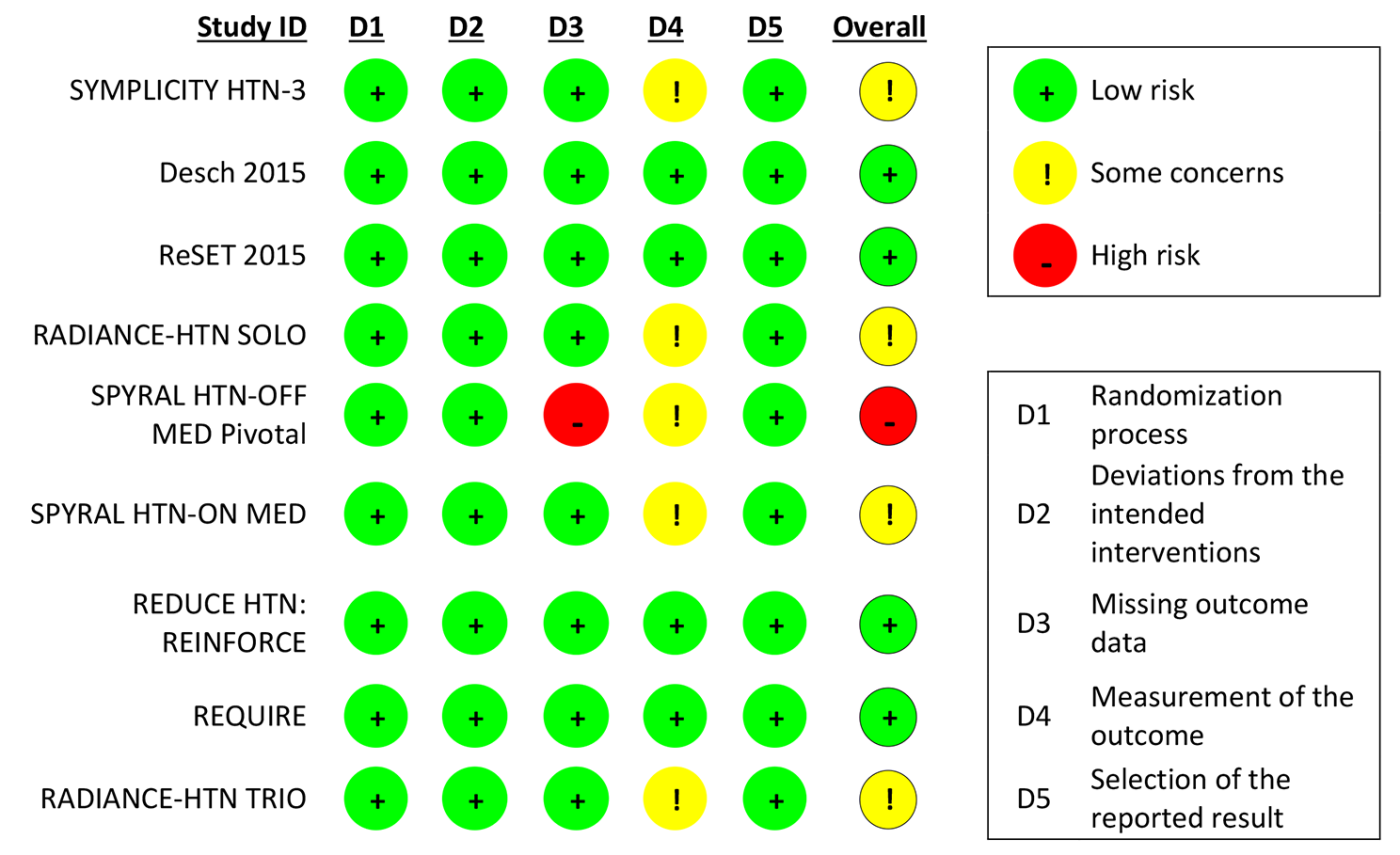
Risk of Bias of individual studies according to the Cochrane Risk of Bias Tool for randomized trials [14]

Risk of bias assessment for each individual study is reported in Fig. 2.

### Office blood pressure monitoring

**Fig. 3:**
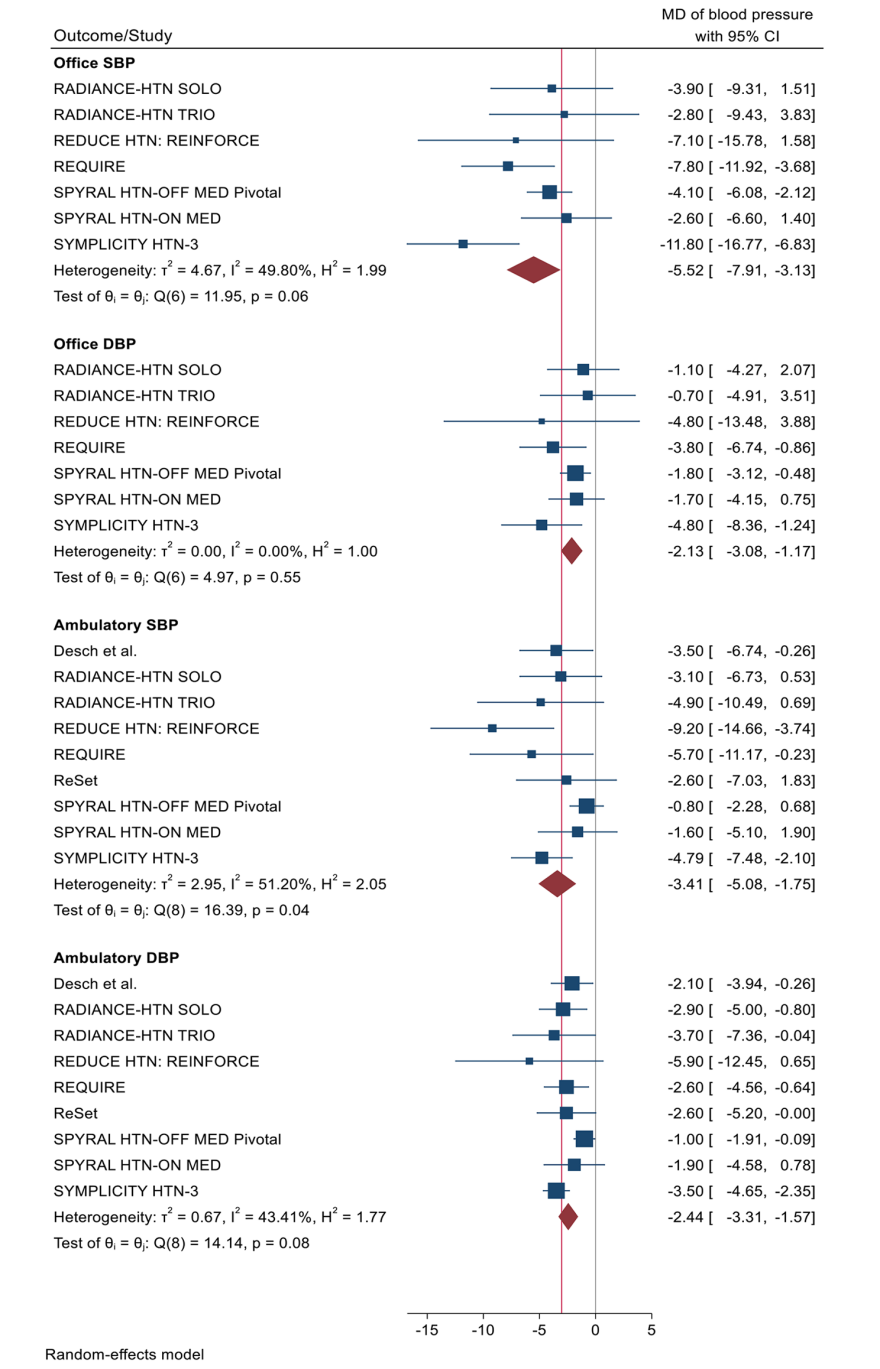
Change in Ambulatory Systolic and Diastolic Blood Pressure and Office Systolic and Diastolic Blood Pressure SBP - Systolic Blood Pressure; DBP – Diastolic Blood Pressure; CI - Confidence Interval; MD - Mean Difference;

Change in office systolic blood pressure at maximum follow-up was available for 7 RCTs and the sham procedure for RDN showed a significant reduction of -5.52 mmHg [95% CI (-7.91, -3.13); P < 0.00001; I^2^ = 49.8%] (Fig. 3).

Change in office diastolic blood pressure at maximum follow-up was available for 7 RCT and the sham procedure for RDN showed a significant reduction of -2.13 mmHg [95% CI (-3.08, -1.17); P < 0.0001; I^2^ = 0%] as shown in (Fig. 3).

The GRADE confidence in this estimate is Low for both outcomes (Table 4).

### Ambulatory blood pressure monitoring

Change in ambulatory (24-hour) mean systolic and diastolic BP at maximum follow-up, was available for 9 RCTs and the sham procedure for RDN showed a reduction of -3.41 mmHg [95% CI (-5.08, -1.75); P < 0.0001; I^2^ = 51.2%] and − 2.44 mmHg [95% CI (-3.31, -1.57); P < 0.00001; I^2^ = 43.41%], respectively (Fig. 3).

The GRADE confidence in this estimate is Very Low for Ambulatory Systolic Blood Pressure and Low for Ambulatory Diastolic Blood Pressure (Table 4).

### Subgroup and other statistical analysis

Jackknife sensitivity analysis assessing the impact of each individual study showed that no study had a significant impact in the pooled estimates when omitted (Additional File Fig. 1).

Regarding subgroup analysis by risk of bias, there was no significant difference between both subgroups (Additional File Fig. 2).

Procedure generation, showed a significant subgroup difference (p = 0.01) only for the outcome change in Office SBP, with a greater magnitude in the SBP reduction for first- (only represented by the SIMPLICITY-HTN trial) vs. second-generation trials (Additional File Fig. 3).

Regarding the random-effects meta-regression, the percentage of diabetic patients and age of patients both showed a correlation with changes in Office SBP and the number of drugs at randomization the percentage of diabetic patients both also showed a correlation with change in Ambulatory DBP (Additional File Fig. 3).

### Procedure-related adverse events

Although most studies only report major adverse events such as death or stroke, 3 studies included data regarding procedure related adverse events. In the first one, the authors reported, in the sham group, 8 patients having pain at the femoral access site and 2 patients with back pain, of a total of 67 patients. Similarly, in the renal denervation group they reported one femoral access site pseudoaneurysm post-procedure treated with thrombin injection, 7 patients with pain at the femoral access site, 4 patients with back pain, and 1 patient with extremity pain, of a total of 69 patients [25]. Another study also reported procedure related pain lasting for > 2 days (e.g., back pain, puncture site pain, etc.), which occurred in 6 patients in each group (RDN and sham group) [26]. In the last study, authors reported 8 patients in each group with procedure-related pain lasting for more than 2 days (RDN – 10,8%; sham – 11,1%) [22].

### Publication bias risk assessment

We also performed a regression-based Egger test for small-study effects to assess potential publication bias in the meta-analysis via funnel plot asymmetry, which showed statistically significant asymmetry for the outcome change in Ambulatory SBP and was not statistically significant for Ambulatory DBP and Office SBP and DBP (Additional File Fig. 4).

The number of studies included in the analysis limits the power of the test. This means that a cautious analysis of the results is required [15].

**Table 1:** Characteristics of trials

First Author/Trial	Year	Total Patients (Sham)	Follow-Up Duration (Months)	Type of Sham	Investigational Device	Primary Efficacy Outcome	Participating Centers	Enrolment Period	Trial Design
SYMPLICITY HTN-3	2014	171	6	Renal Angiography	Symplicity Renal Denervation System (Medtronic)	Mean change in office systolic blood pressure from baseline to 6 months	88 sites in the United States	October 2011 to May 2013	Prospective, single-blind, randomized, sham-controlled trial
Desch et al.	2015	36†/35‡	6	Renal Angiography	Symplicity Flex Cathether (by Ardian Inc, Palo Alto, CA, USA)	Change in 24-hour systolic blood pressure at 6 months	Single-center, Leipzig, Germany	July 2012 to January 2014	Sham-controlled, randomized, single-center trial
ReSet	2016	33†/32‡	3	Radiograph scan while the patient was sedated	Symplicity Renal Denervation Catheter (Medtronic)	Mean change in daytime systolic ambulatory blood pressure measurements from baseline to 3 months	Single-center, Skejby, Denmark	No information	Sham-controlled, double-blind, randomized single-center trial
RADIANCE-HTN SOLO	2018	72	2	Renal Angiography	Paradise endovascular ultrasound renal denervation system	Mean change in daytime ambulatory systolic blood pressure from baseline to 2 months	21 centers in the USA and 18 in Europe	March 28, 2016 to December 28, 2017	Multicenter, single-blind, randomized, sham-controlled trial
SPYRAL HTN-OFF MED Pivotal	2020	165†/ 137*/130**	3	Renal Angiography	Symplicity Spyral multielectrode renal denervation catheter^#^ and Symplicity G3 radiofrequency Generator^$^	Change in mean 24-h systolic blood pressure from baseline to 3 months	44 centers in Australia, Austria, Canada, Germany, Greece, Ireland, United Kingdom and United States of America	June 25, 2015 to October 15, 2019	Prospective, single-blinded, sham-control trial
SPYRAL HTN-ON MED	2018	42† / 39‡	6	Renal Angiography	Symplicity Spyral multielectrode renal denervation catheter^#^ and Symplicity G3 radiofrequency generator^$^	Change in ambulatory blood pressure from baseline to 6 months	25 centers in the USA, Germany, Japan, UK, Australia, Austria and Greece	July 22, 2015 to June 14, 2017	Randomized, Single-blind, sham-control, proof-of-concept trial
REDUCE HTN: REINFORCE	2020	17*/15**	2 (8 weeks)	Renal Angiography	Vessix Renal Denervation system	Mean reduction in average 24-h ambulatory systolic blood pressure by 8 weeks post-randomization	12 centers in the United States	April 2015 to October 2017	Prospective, multicenter, single blinded, randomized, controlled, pilot trial
REQUIRE	2021	71†/ 66*/67**	3	Renal Angiography	Paradise endovascular ultrasound renal denervation system	Change in 24-hour ambulatory systolic blood pressure from baseline to 3 months	53 centers in Japan and Korea	January 12, 2017 to March 31, 2020	Multicenter, single-blind, randomized, sham-controlled trial
RADIANCE-HTN TRIO	2021	67	2	Renal Angiography	Paradise endovascular ultrasound renal denervation system	Change in daytime ambulatory systolic blood pressure from baseline to 2 months	28 centers in Europe and 28 centers in USA	March 11, 2016 to March 13, 2020	Multicenter, single-blind, randomized, sham-controlled trial

† Randomized patients; ‡ Patients included in iTT analysis for both the outcomes “change in office blood pressure” and “change in 24 h ambulatory blood pressure”; * Patients included in iTT analysis for the outcome “change in office blood pressure”; ** Patients included in iTT analysis for the outcome “change in 24 h ambulatory blood pressure.”; # Medtronic; Galway, Ireland); $ (Medtronic; Minneapolis, MN, USA)

**Table 2:** Baseline patient characteristics in sham arm of included randomized clinical trials

	SYMPLICITY HTN-3	Desch et al.	ReSet	RADIANCE-HTN SOLO	SPYRAL HTN-OFF MED Pivotal	SPYRAL HTN-ON MED	REDUCE HTN: REINFORCE	REQUIRE	RADIANCE-HTN TRIO
**Number of patients**	171	36	33	72	165	42	17	67	67
**Age - n ± SD**	56.2 ± 11.2	57.4 ± 8.6	57.1 ± 6.6	53.8 ± 10	52.6 ± 10.4	53 ± 10.7	58.2 ± 9.8	55.6 ± 12.1	52.8 ± 9.1
**Gender (male) - n (%)**	110 (64.3)	25 (69)	(73)	39 (54)	113 (68)	34 (81)	13 (76)	53 (79.1)	53 (79)
**Race - n (%)**									
white	119 (69.6)	36 (100)	(97)	52 (72)	50 (30)	15 (36)	14 (82)	NR	50 (75)
black	50 (29.2)	0	NR	13 (18)	31 (19)	5 (12)	3 (18)	NR	12 (19)
other	2 (1.2)	0	NR	7 (10)	5 (3) Δ	1 (2) ¥	1 (6)	NR	3 (4.5) ‡
**Comorbidities - n (%)**									
Chronic Kidney Disease - GFR < 60	17 (9.9)	NR *	(18)	3 (4)	NR	NR	NR **	18 (26.9)	7 (11)
Diabetes Mellitus 2	70 (40.9)	13 (36)	(31)	5 (7)	9 (5)	8 (19)	2 (12)	20 (29.9)	17 (25)
Obstructive Sleep Apnea	54 (31.6)	NR	(12)	8 (11)	12 (7)	10 (24)	1 (6)	8 (11.9)	11 (16)
Peripheral Artery Disease	5 (2.9)	2 (6)	NR	NR	0	0	NR	2 (3)	NR
Coronary Artery Disease	43 (25.1)	17 (47)	(15)	NR	8 (5)	1 (2)	3 (18)	9 (13.4)	NR
**Smokers - n (%)**	21 (12.3)	4 (11)	(15)	NR	27 (16)	11 (26)	2 (12)	NR	NR
**Hyperlipidemia - n (%)**	111 (64.9)	NR	NR	NR	NR	NR	4 (24)	40 (59.7)	NR
**BMI (kg/m** ^**2**^ **) - n ± SD**	33.9 ± 6.4	31.2 ± 4.6	28.8 ± 3.9	29.0 ± 5.0	30.9 ± 5.5	32.5 ± 4.6	NR	28.4 ± 4.5	32.6 ± 5.4
**Mean number of antihypertensive agents - n ± SD**	5.2 ± 1.4	4.3 ± 1.3	4.2 ± 1.1	0 (OFF MED)	0 (OFF MED)	2.3 ± 0.8	0 (OFF MED)	NR	NR

Values are n ± SD or n (%). SD – Standard Deviation; NR – not reported; BMI – Body Mass Index; GFR – Glomerular Filtration Rate

Δ Not reported for 79 (48) patients; ¥ Not reported for 20 (48) patients; ‡ Not reported for 2 (3) patients; * Only reported mean GFR − 84 ± 20 ml/min/1.73 m^2^; ** Only reported mean GFR − 86.2 ± 16.2 ml/min/1.73 m^2^

**Table 3:** Blood pressure reduction (mmHg) in sham arm of included trials

	SYMPLICITY HTN-3	Desch et al.	ReSet	RADIANCE-HTN SOLO	SPYRAL HTN-OFF MED Pivotal	SPYRAL HTN-ON MED	REDUCE HTN: REINFORCE	REQUIRE	RADIANCE-HTN TRIO
Sham Group									
Reduction in Ambulatory SBP	-4.79	-3.5	-2.6	-3.1	-0.8	-1.6	NR	-5.7	NR
Reduction in Ambulatory DBP	-3.1	-2.1	-2.6	-3	-1.0	-1.9	NR	-2.6	NR
Reduction in Office SBP	-11.74	NR	NR	-3.9	-4.1	-2.6	-7.1	-7.8	-2.8
Reduction in Office DBP	-4,6	NR	NR	-1.2	-1.8	-1.7	-4.8	-3.8	-0.7

Values are mmHg. DBP – Diastolic Blood Pressure; NR – Not Reported; RDN – Renal Denervation; SBP – Systolic Blood Pressure

**Table 4:** Certainty of Evidence of sham intervention for renal denervation compared to baseline values for adult patients with hypertension

Certainty assessment						
Participants (studies)	Risk of bias	Inconsistency	Indirectness	Imprecision	Publication bias	Overall certainty of evidence
**Ambulatory Systolic Blood Pressure**						
637 (9 Pre-post data RCTs)	serious^a^	serious^b^	not serious	not serious	publication bias strongly suspected^c^	$\oplus$◯◯◯ Very low
**Ambulatory Diastolic Blood Pressure**						
637 (9 Pre-post data RCTs)	serious^a^	serious^d^	not serious	not serious	none	$$\oplus$$$$\oplus$$◯◯ Low
**Office Systolic Blood Pressure**						
570 (7 Pre-post data RCTs)	serious^a^	serious^e^	not serious	not serious	none	$$\oplus$$$$\oplus$$◯◯ Low
**Office Diastolic Blood Pressure**						
570 (7 Pre-post data RCTs)	serious^a^	not serious	not serious	not serious	none	$$\oplus$$$$\oplus$$◯◯ Low

RCTs: randomized clinical trials

**a.** Five trials were considered as low risk of bias, while 3 trials had some concerns regarding outcome measurement of the outcome since the trial funder was in charge of data collection and management. 1 trial was considered to have a high risk of bias, due to missing outcome data that may be dependent on its true value

**b.** Heterogeneity (i^2^) = 51.2%; **c.** Publication bias was assessed via funnel plot asymmetry, which was statistically significant (P = 0.0016); **d.** Heterogeneity (i^2^) = 43.41%;

**e.** Heterogeneity (i^2^) = 49.8%;

## Discussion

In this meta-analysis including data from 9 RCTs we found that the sham procedure for RDN showed a significant decrease in ambulatory systolic and diastolic blood pressure of -3.41 mmHg and − 2.44 mmHg, respectively as well as in decreasing Office Systolic and Diastolic Blood Pressure by -5.52 mmHg and − 2.13 mmHg, respectively, in patients with hypertension.

Recently published systematic reviews with meta-analysis comparing the BP lowering efficacy of RDN versus sham effect, also reported results in accordance with this meta-analysis, all of them showing a mild but significant reduction in Office or Ambulatory BP in patients submitted to sham procedure of RDN [30–33].

Moreover, RCTs assessing the BP lowering effects of RDN without sham procedures showed a more pronounced BP reduction than sham-controlled RCTs, which evidences a possible misattribution of a placebo effect as a treatment effect in no sham-controlled trials [33]. This corroborates the results of our meta-analysis, that placebo effect plays an important role in the RDN denervation surgery and therefore might have an impact in renal nerve pathophysiology and the development of hypertension. The random-effects meta-regression performed in our study also showed a correlation between number of diabetic patients and change in Office SBP and Ambulatory DBP, which may be related to some degree of renal autonomic disfunction, even though the correlation was not present for the other two measured outcomes.

Regarding the placebo effect in BP, research conducted in this area suggests that the placebo effect might decrease BP by modulating the ANS [34–39], thereby acting on the same pathophysiological pathways as the catheter-based RDN being studied for the treatment of hypertension. While some studies have shown that the expectation of lowering BP through verbal suggestion has in fact led to an effective decrease in SBP, [35–38] the same effect has not been reported consistently [39].

Interestingly, unlike our results that showed a decrease in both systolic and diastolic BP induced by sham effect, some studies found that placebo interventions along with verbal suggestion of blood-pressure lowering reduced systolic but not diastolic BP [38]. Considering that systolic blood pressure is largely determined by cardiac output, while diastolic blood pressure is mainly influenced by peripheral vascular resistance, while both are still regulated primarily by the SNS, these results also provide initial evidence for a possible organ-specific patterned placebo effect in the cardiovascular system. This divergence in results also evidences that the BP lowering effect mediated through verbal suggestion, placebo drugs or sham interventions may be achieved through different pathways and requires further investigation [38].

On another note, regarding the efficacy of the placebo effect in pain-related conditions, some studies showed that more invasive placebos were associated with a larger placebo response than for example oral placebos, probably due to enhanced expectation toward invasive procedures [12]. Therefore, the same mechanism may also account for the results found in this review, since the sham intervention can be considered one of the most invasive interventions possible (with the patients being under anesthesia and sedation).

In this light and considering the results of this meta-analysis, there is still much to be addressed in understanding the biological basis of placebo and the sham effect in renal sympathetic afferent and efferent nerves, in the cross talk between the kidney and the central nervous system and even in cardiovascular pathophysiology in general. Further research in this area may help investigators reach a more extensive understanding of the key mechanisms in the progression of hypertension as well as predictors of blood pressure response to both renal denervation surgery and sham interventions.

Considering current standard of care for the management of hypertension, several different lifestyle changes and pharmacological treatments are recommended. Among these, salt reduction is almost a consensual recommendation worldwide [1, 40]. It is interesting to notice that salt reduction has approximately the same effect in lowering BP as the sham effect of RDN that we found in the current review, with 4.4 g of salt reduction in hypertensive patients showing a decrease of -5.39 mmHg in SBP and a decrease of -2.82 mmHg in DBP [41].

Previous studies have described that every 10 mmHg reduction in SBP significantly reduced the risk of major cardiovascular disease events (relative risk [RR] 0.80, 95% CI 0.77–0.83), coronary heart disease (0.83, 0.78–0.88), stroke (0.73, 0.68–0.77), and heart failure (0.72, 0.67–0.78), which led to a significant 13% reduction in all-cause mortality (0.87, 0.84–0.91) [42]. A recent meta-analysis showed that a reduction in 5 mmHg of systolic blood pressure was associated with a significant reduction in major cardiovascular event rate (HR 0.91, 95% CI 0.89–0.94) [43].

Considering the high burden that cardiovascular diseases and especially hypertension pose on healthcare systems, addressing these questions is of extreme importance for developing better health policies supported by evidence-based medicine. As also seen in coronary disease, [44] sham procedures are important to address efficacy issue of invasive interventions in cardiovascular diseases or risk factors, particularly if outcomes can be modulated by expectations [44].

### Limitations

Since we evaluated the effect of BP reduction within the sham arm of RCTs, therefore the control group of the trials, a major limitation in the present systematic review is the absence of an adequate control group. A suitable control group would be extremely relevant in order to distinguish an effect of the placebo intervention from confounding factors, such as the natural history of arterial hypertension investigation or regression to the mean [45].

On another hand, the availability of a small number of randomized sham-controlled trials for RDN is a strong limitation, having therefore a small sample for inclusion in the meta-analysis. The short follow-up time of patients submitted to this procedure represents another limitation, especially since unblinding of the intervention occurred between 8 weeks and 12 months, having an impact in the placebo effect and long-term conclusions, especially if the placebo effect would decline with time. All these factors account for a possible high level of bias when interpreting the results of post-hoc analysis.

In addition, no direct measurement method was used to evaluate the degree of RDN in all included trials, so it remains unclear what is the true mechanism behind the BP lowering effect of the sham intervention: whether it induced some degree of functional RDN or if it worked through an entirely different pathway.

Another limitation is related to the “Hawthorne effect” or “observer effect” which describes a change in normal behavior when individuals are aware they are being observed. This effect may be present in almost all clinical trials and may have an impact on effect estimates. Nevertheless, many included studies had a run-in period, which means patients were monitored for a specific period of time and used the BP values measured after this time as baseline, possibly neutralizing the Hawthorne Effect [10, 20, 22, 24–26].

Lastly, our results are also subject to some limitations inherent to the meta-analysis methodology, integrating data from different clinical trials with different included populations and study protocols.

## Conclusion

Despite recent data indicating that RDN might be an effective treatment for patients with resistant hypertension when compared to a sham intervention, our results indicate that the sham intervention for RDN also has a significant effect on lowering Office and Ambulatory (24-h) Blood Pressure in adult patients with hypertension. This highlights that BP itself might be sensitive to placebo-like effect and also brings further difficulties in establishing the BP lowering efficacy of invasive interventions due to the magnitude of the sham effect.

## Electronic supplementary material

Below is the link to the electronic supplementary material.

Additional File Table 1: Search strategies. Additional File Table 2: Blinding Index of patients in included trials. Additional File Table 3: Metaregression according to age, male (%), diabetic patients (%), number of anti-hypertensive drugs. Additional File Figure 1: Jackknife leave-one-out sensitivity analysis. Additional File Figure 2: Subgroup analysis according to risk of bias. Additional File Figure 3: Subgroup analysis according to generation of the procedure. Additional File Figure 4: Funnel plot for primary outcomes.

## Data Availability

All data generated or analysed during this study are included in this published article and its supplementary information files.
