# Supplementary material for: The effect of catheter-based sham renal denervation in hypertension: systematic review and meta-analysis
Source: BMC Cardiovasc Disord. 2023 May 12;23:249. doi: 10.1186/s12872-023-03269-w (PMC10182607; doi:10.1186/s12872-023-03269-w)

Supplementary Material

Table of Contents

Supplementary Table 1 – Search strategies 2
Supplementary Table 2. Blinding Index of patients in included trials 3
Supplementary Table 3. Metaregression according to age, male (%), diabetic patients (%), ... 4
number of anti-hypertensive drugs 4
Supplementary Figure 1. Jackknife leave-one-out sensitivity analysis 5
Supplementary Figure 2. Subgroup analysis according to risk of bias. 7
Supplementary Figure 3. Subgroup analysis by generation of procedure 9
Supplementary Figure 4. Funnel plot for primary outcomes 11

Supplementary Table 1 – Search strategies

#	Searches
**1**	(renal adj2 denervation).af.
**2**	randomized controlled trial.pt.
**3**	controlled clinical trial.pt.
**4**	randomized.ab.
**5**	sham.ab.
**6**	randomly.ab.
**7**	trial.ab.
**8**	exp animals/ not humans.sh.
**9**	2 or 3 or 4 or 5 or 6 or 7
**10**	9 not 8
**11**	1 and 10
**12**	sham.af.
**13**	sham*.af.
**14**	sham-control*.af.
**15**	12 or 13 or 14
**16**	11 and 15
**17**	remove duplicates from 16

Supplementary Table 2. Blinding Index of patients in included trials

		SYMPLICITY HTN-3	Desch et al.	ReSet	RADIANCE-HTN SOLO		SPYRAL HTN-OFF MED Pivotal	SPYRAL HTN-ON MED	REDUCE HTN: REINFORCE	RE[illegible]
James Blinding Index *	Discharge	0,68 [0,64; 0,72]	-	0,83	0,7 [0,62; 0,77]		0,66 [0,61; 0,71]	0,78 [0,70; 0,85]	0,64 [0,51; 0,78]	[illegible]
	Maximum follow-up	0,77 [0,74; 0,81]	-	-	0,6 [0,52; 0,68]		0,53 (0,48; 0,59)	0,64 [0,54; 0,74]	0,56 [0,42; 0,70]	[illegible]
Bang Blinding Index **					**RSD**	**Sham**				
	Discharge	-	-	-	0,30 [0,16; 0,44]	-0·01 [-0,17; 0,14]	-	-	-	[illegible]
	Maximum follow-up	-	-	-	0,24 [0,08; 0,41]	0,18 [0,01; 0,35]	-	-	-	[illegible]

RSD – Renal Sympathetic Denervation

* Confidence intervals superior to and excluding 0,50 indicate effective blinding.[28]

** Values approaching zero indicate effective blinding. Values approaching either 1 or -1 indicate incomplete blinding.[29]

Supplementary Table 3. Metaregression according to age, male (%), diabetic patients (%), number of anti-hypertensive drugs

Variable	Coefficient	Standard error	P>z	[95% conf.	interval]
**Ambulatory SBP**					
Age	-.2824554	0.4196696	0.501	-1.104993	.5400819
Male (%)	-.0403876	.1119416	0.718	-.259789	.1790139
Diabetes (%)	-.0729278	.0504913	0.149	-.171889	.0260333
Drugs	-.2166692	.4170695	0.603	-1.03411	.6007721
**Ambulatory DBP**					
Age	-.3167957	.1807991	0.080	-.6711554	.0375641
Male (%)	.0108073	.0601088	0.857	-.1070038	.1286184
Diabetes (%)	-.0543901	.0185257	0.003	-.0906999	-.0180803
Drugs	-.3277363	.1495818	0.028	-.6209113	-.0345613
**Office SBP**					
Age	-1.731316	.5721542	0.002	-2.852717	-.609914
Male (%)	.063492	.148603	0.669	-.2277646	.3547485
Diabetes (%)	-.1598503	.0654515	0.015	-.2881328	-.0315677
Drugs	-1.154066	.6512575	0.076	-2.430508	.122375
**Office DBP**					
Age	-.7335358	.4019761	0.068	-1.521395	.054323
Male (%)	-.0182236	.0632087	0.773	-.1421103	.1056631
Diabetes (%)	-.0662281	.0417557	0.113	-.1480678	.0156117
Drugs	-.4408458	.333437	0.186	-1.09437	.2126787

SBP – Systolic Blood Pressure; DBP – Diastolic Blood Pressure; Conf. interval – Confidence interval

Supplementary Figure 1. Jackknife leave-one-out sensitivity analysis

(A) Change in Ambulatory Systolic Blood Pressure

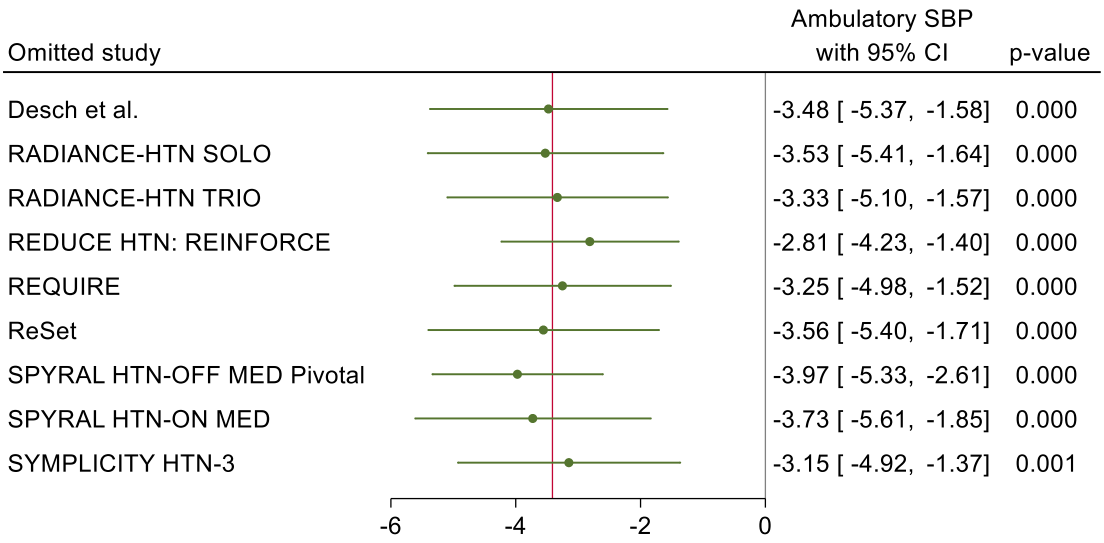

(B) Change in Ambulatory Diastolic Blood Pressure

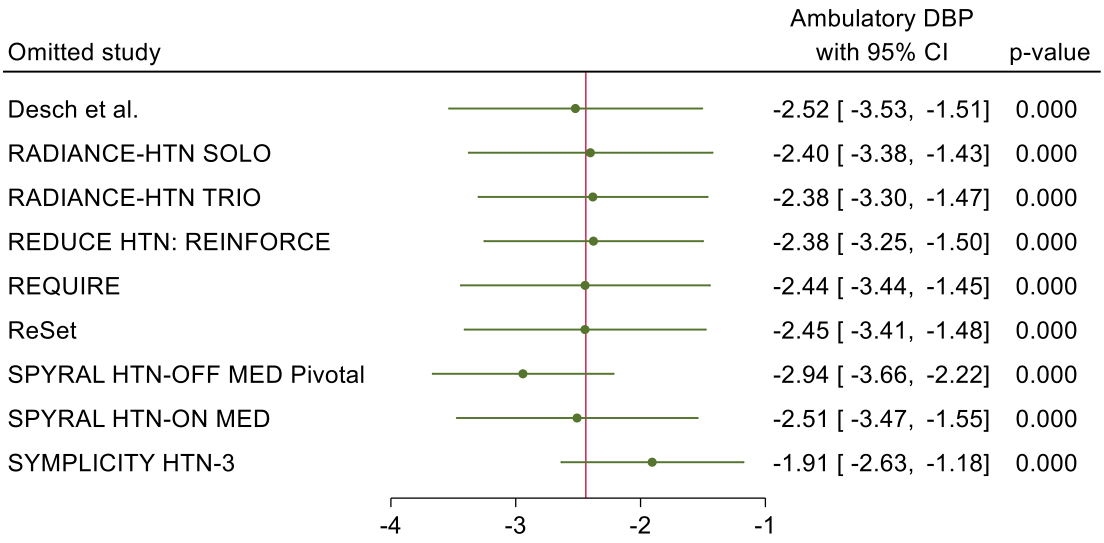

(C) Change in Office Systolic Blood Pressure

Omitted study	Office SBP with 95% CI	p-value
RADIANCE-HTN SOLO	-5.80 [-8.55, -3.05]	0.000
RADIANCE-HTN TRIO	-5.84 [-8.48, -3.20]	0.000
REDUCE HTN: REINFORCE	-5.44 [-8.03, -2.85]	0.000
REQUIRE	-5.09 [-7.74, -2.43]	0.000
SPYRAL HTN-OFF MED Pivotal	-6.02 [-9.12, -2.92]	0.000
SPYRAL HTN-ON MED	-6.14 [-8.87, -3.41]	0.000
SYMPLICITY HTN-3	-4.39 [-5.88, -2.89]	0.000

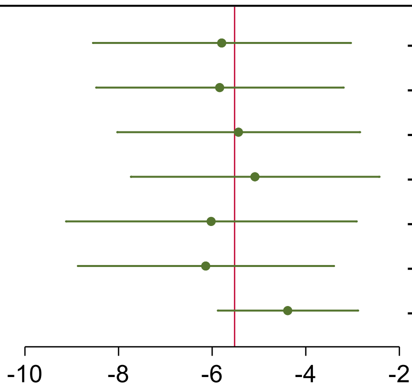

(D) Change in Office Diastolic Blood Pressure

Omitted study	Office DBP with 95% CI	p-value
RADIANCE-HTN SOLO	-2.23 [-3.23, -1.23]	0.000
RADIANCE-HTN TRIO	-2.20 [-3.18, -1.23]	0.000
REDUCE HTN: REINFORCE	-2.09 [-3.05, -1.14]	0.000
REQUIRE	-1.93 [-2.94, -0.92]	0.000
SPYRAL HTN-OFF MED Pivotal	-2.48 [-3.85, -1.11]	0.000
SPYRAL HTN-ON MED	-2.20 [-3.24, -1.17]	0.000
SYMPLICITY HTN-3	-1.92 [-2.91, -0.93]	0.000

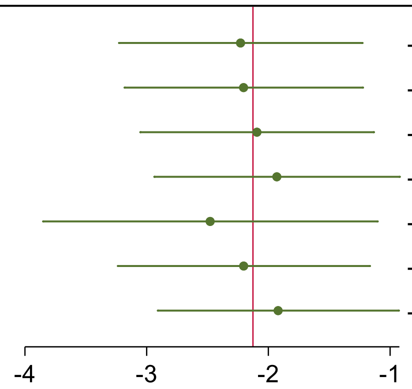

SBP – Systolic Blood Pressure; DBP – Diastolic Blood Pressure; CI – Confidence Interval

Supplementary Figure 2. Subgroup analysis according to risk of bias.

(A) Change in Ambulatory Systolic Blood Pressure (SBP)

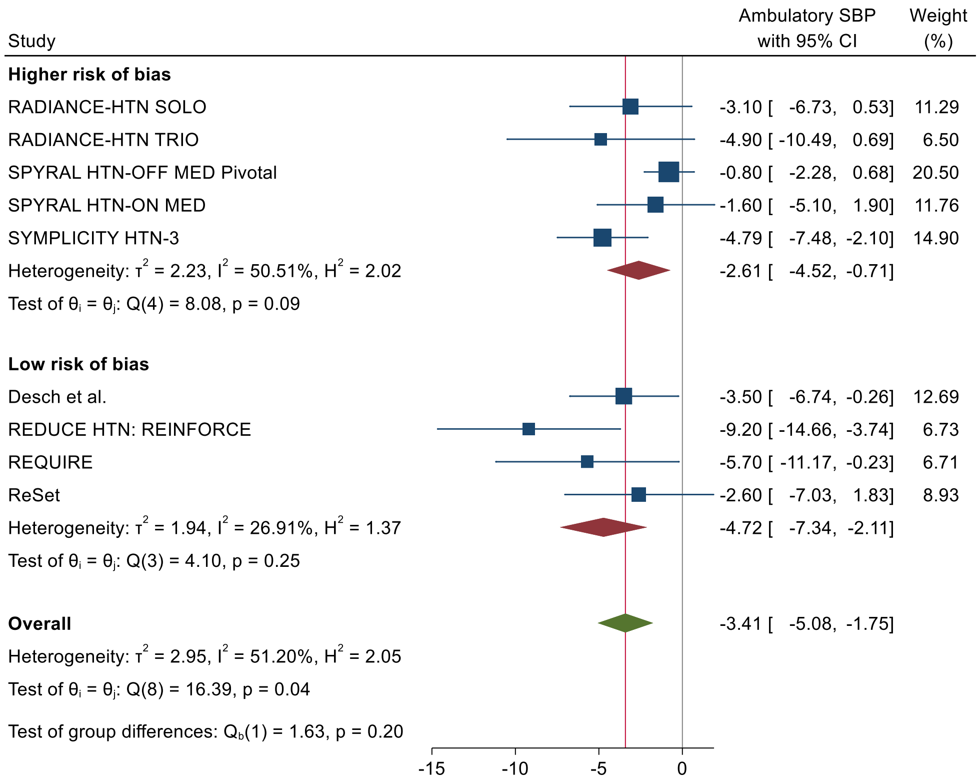

(B) Change in Ambulatory Diastolic Blood Pressure (DBP)

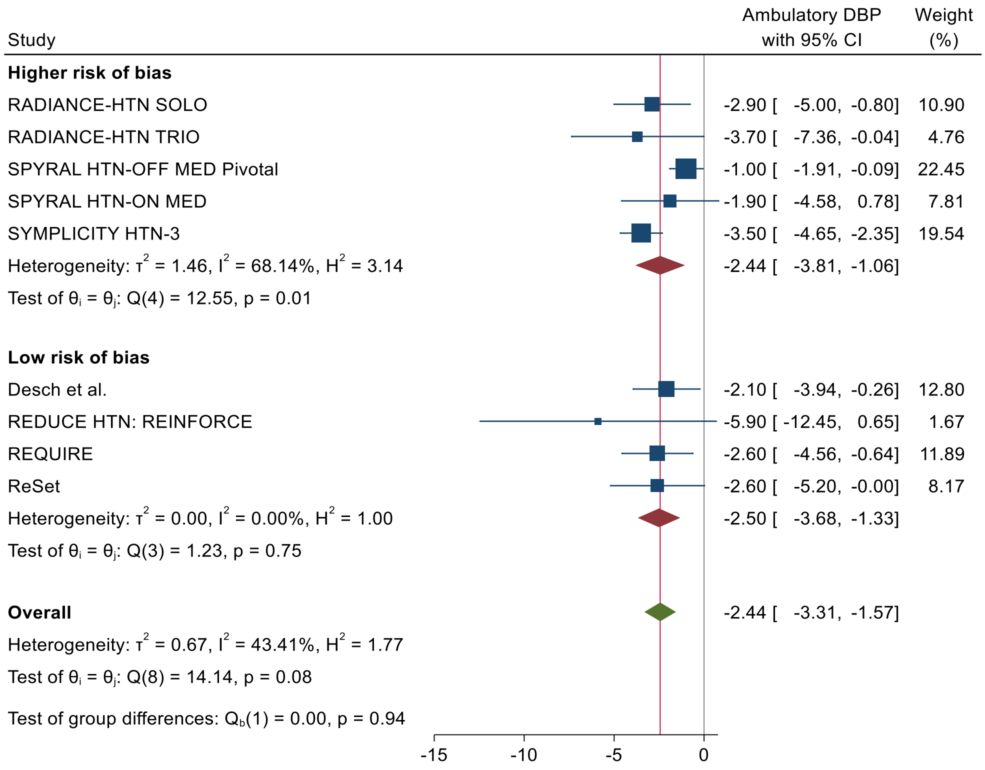

(C) Change in Office Systolic Blood Pressure (SBP)

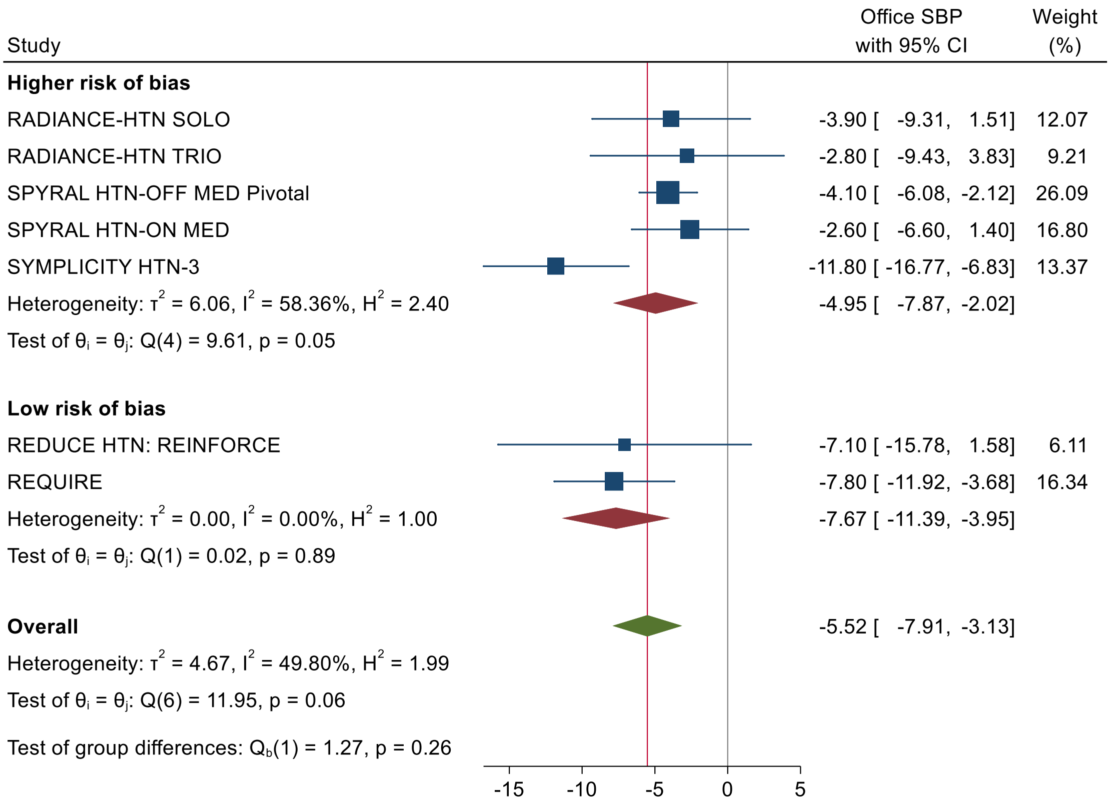

(D) Change in Office Diastolic Blood Pressure (DBP)

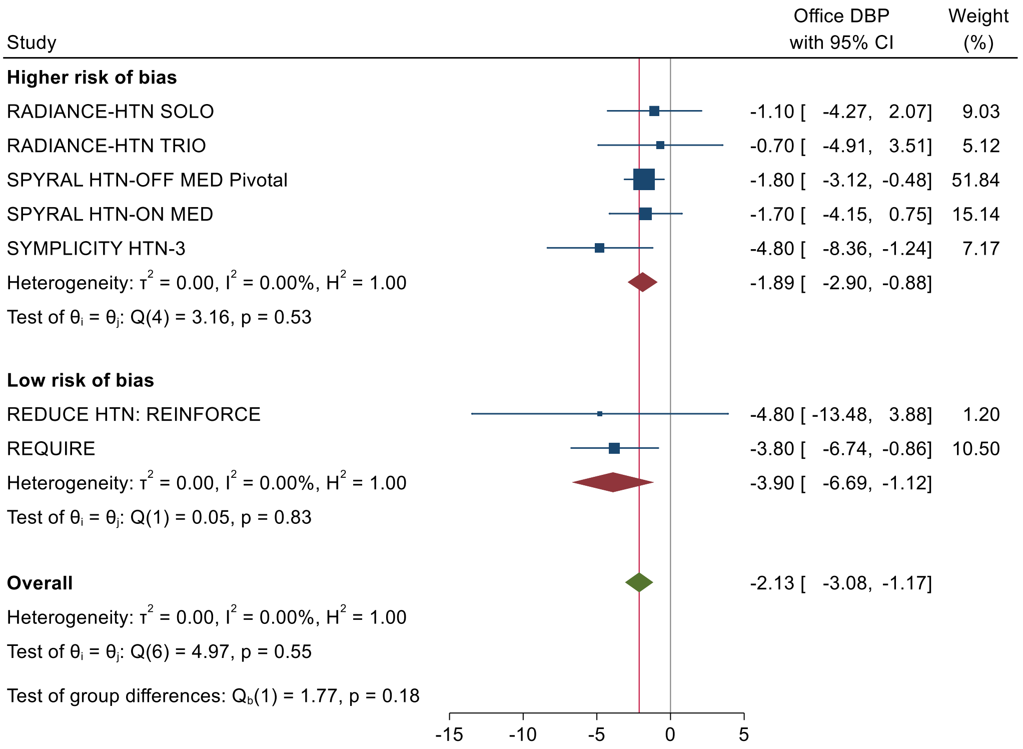

SBP – Systolic Blood Pressure; DBP – Diastolic Blood Pressure; CI – Confidence Interval

Supplementary Figure 3. Subgroup analysis by generation of procedure

(A) Change in Ambulatory Systolic Blood Pressure

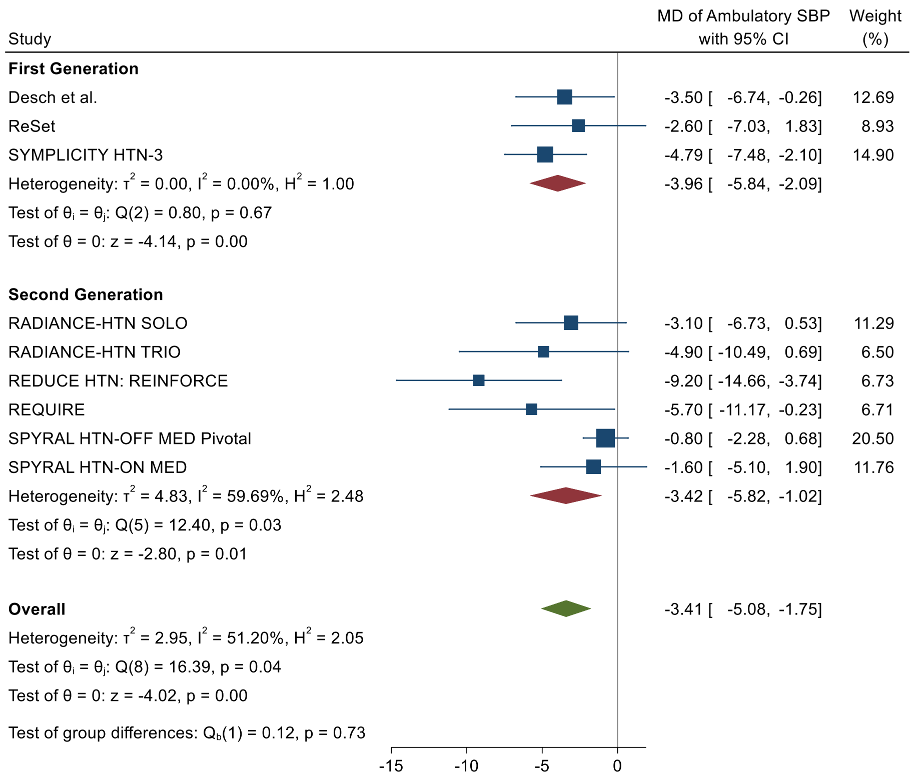

(B) Change in Ambulatory Diastolic Blood Pressure

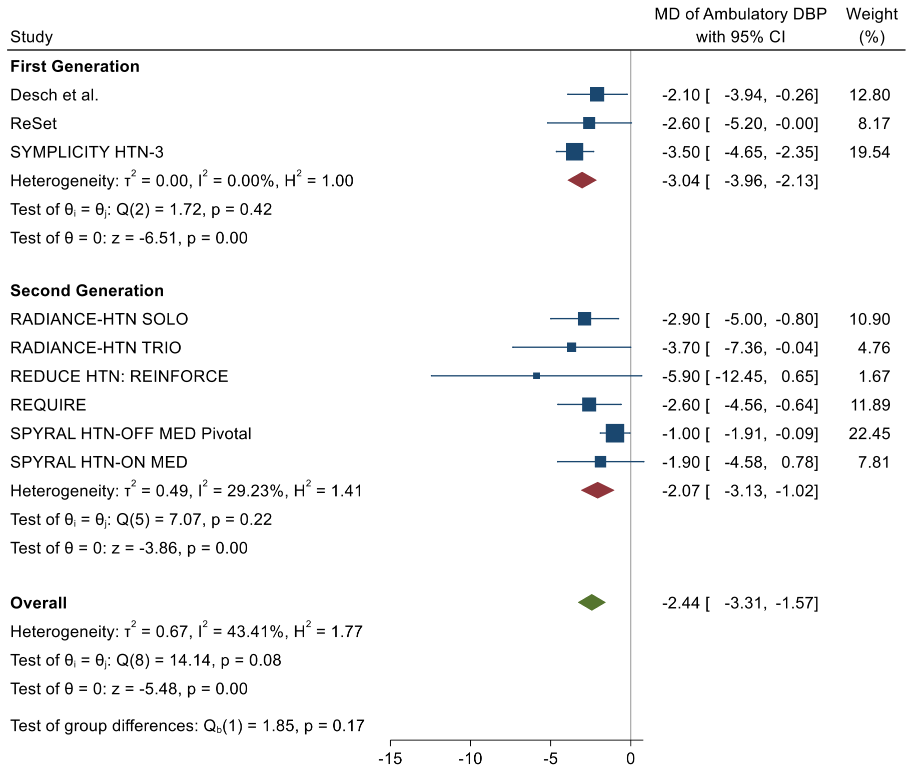

(C) Change in Office Systolic Blood Pressure

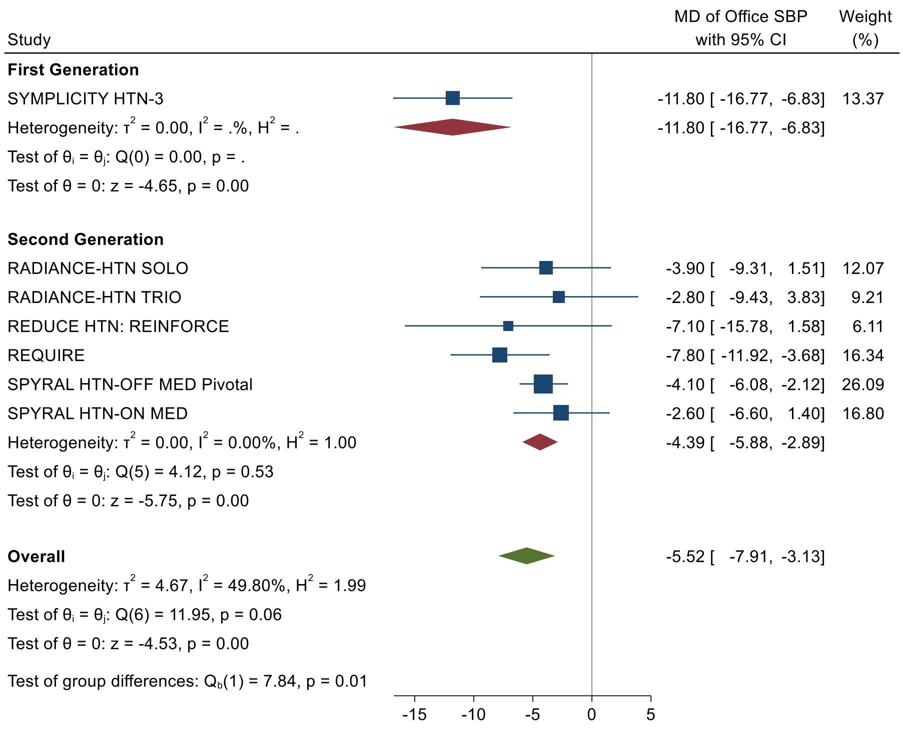

(D) Change in Office Diastolic Blood Pressure

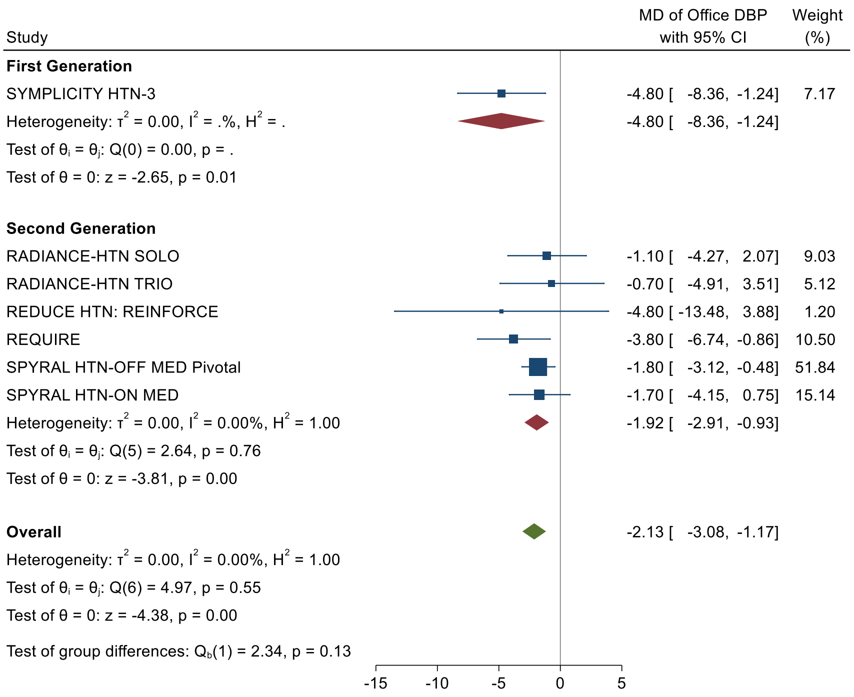

BP - Blood Pressure; RD - Renal Denervation; CI - Confidence Interval; IV - Inverse Variance;

Supplementary Figure 4. Funnel plot for primary outcomes

(A) Ambulatory Systolic Blood Pressure (P = 0.0016)

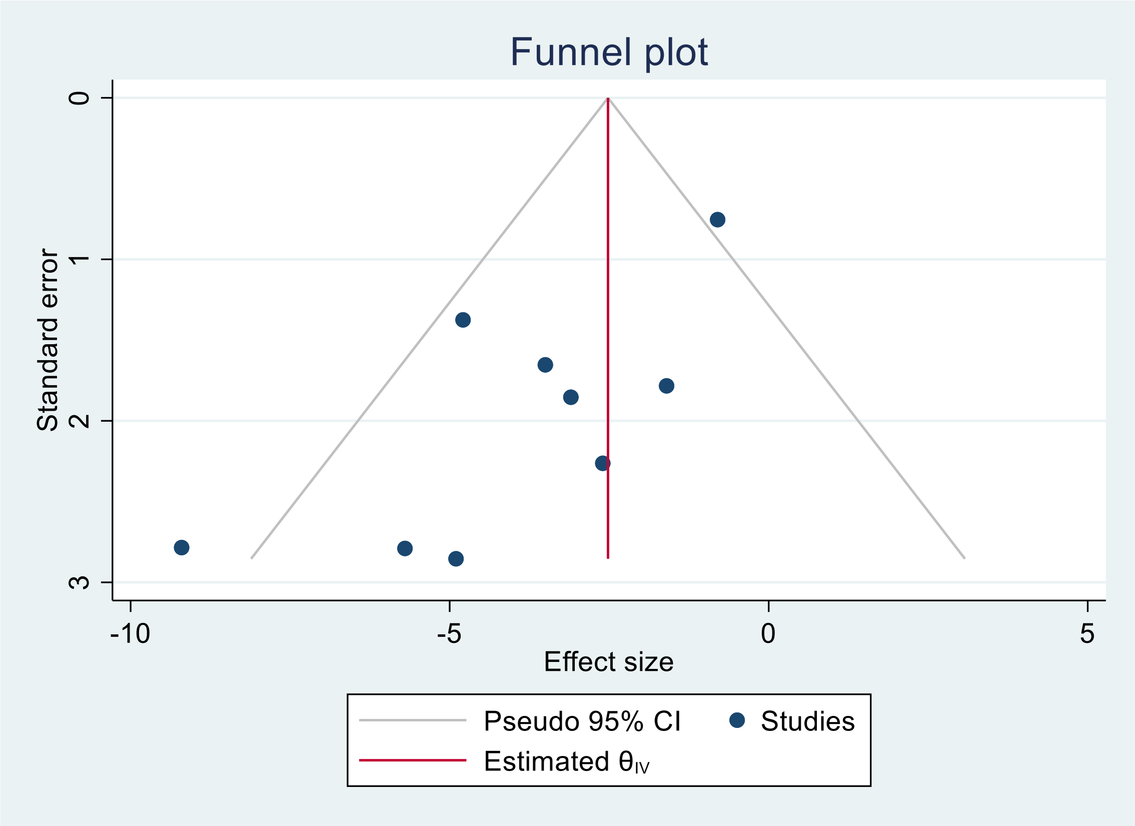

(B) Ambulatory Diastolic Blood Pressure (P = 0.1900)

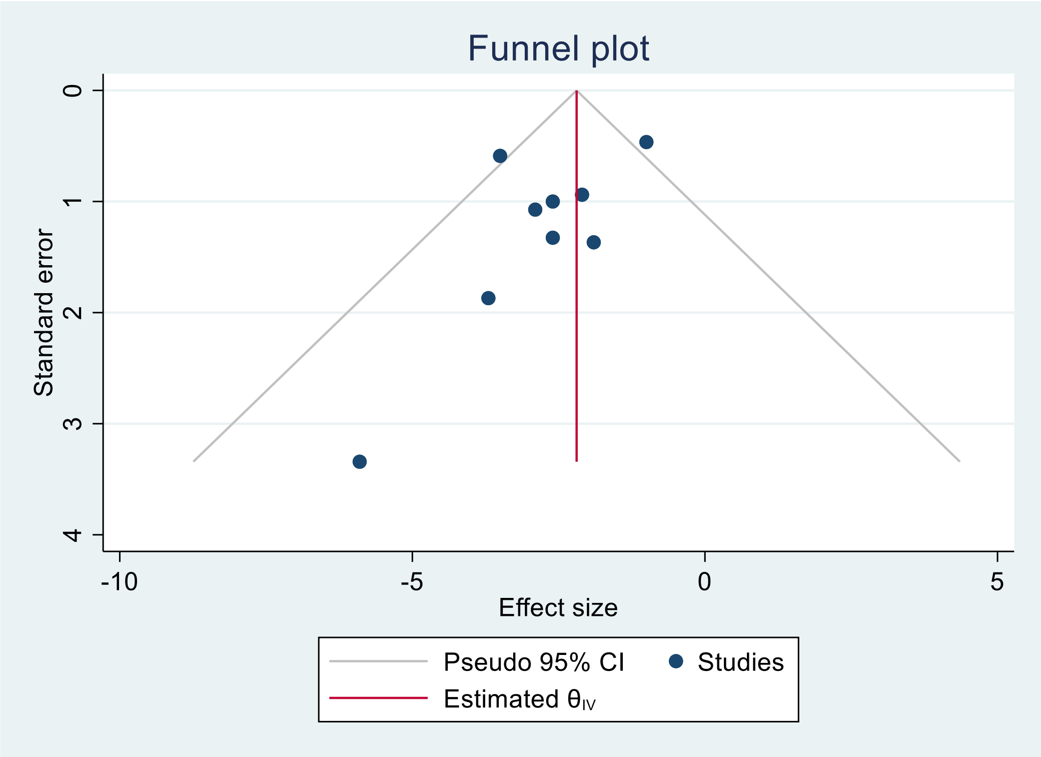

(C) Funnel plot for Office Systolic Blood Pressure (P = 0.6677)

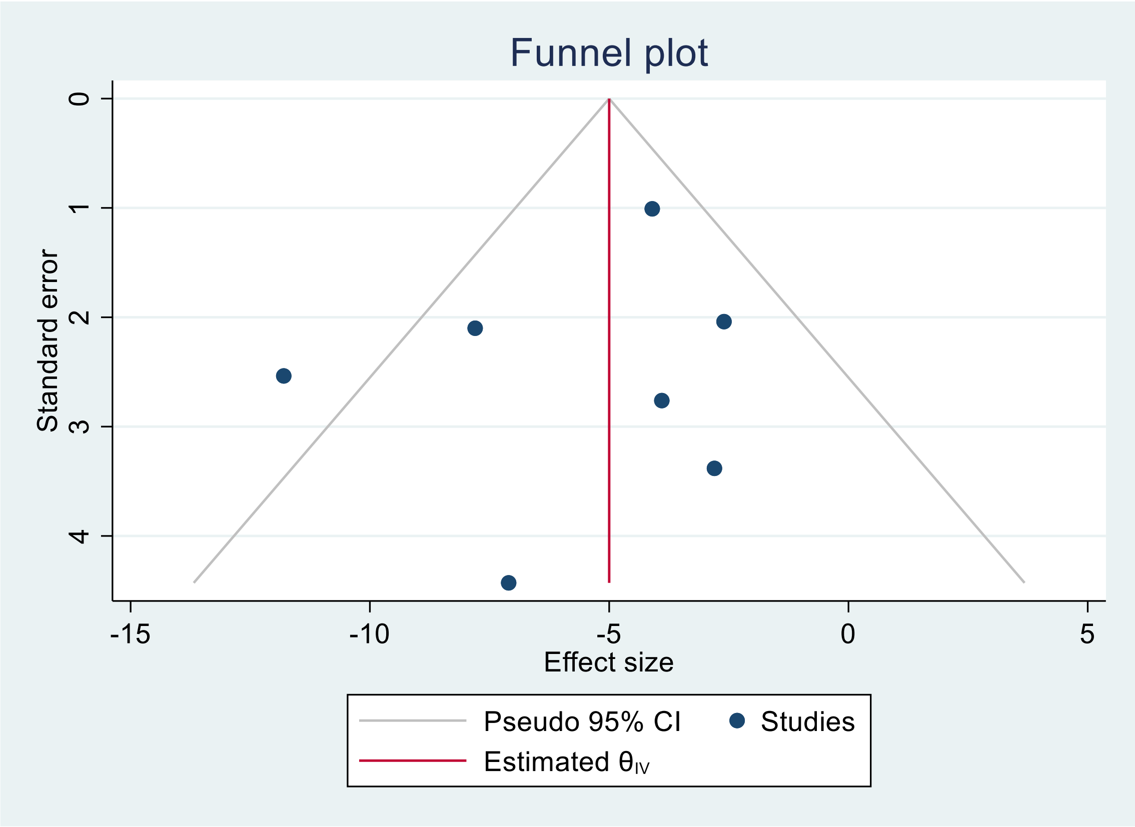

(D) Funnel plot for Office Diastolic Blood Pressure (P = 0.3889)

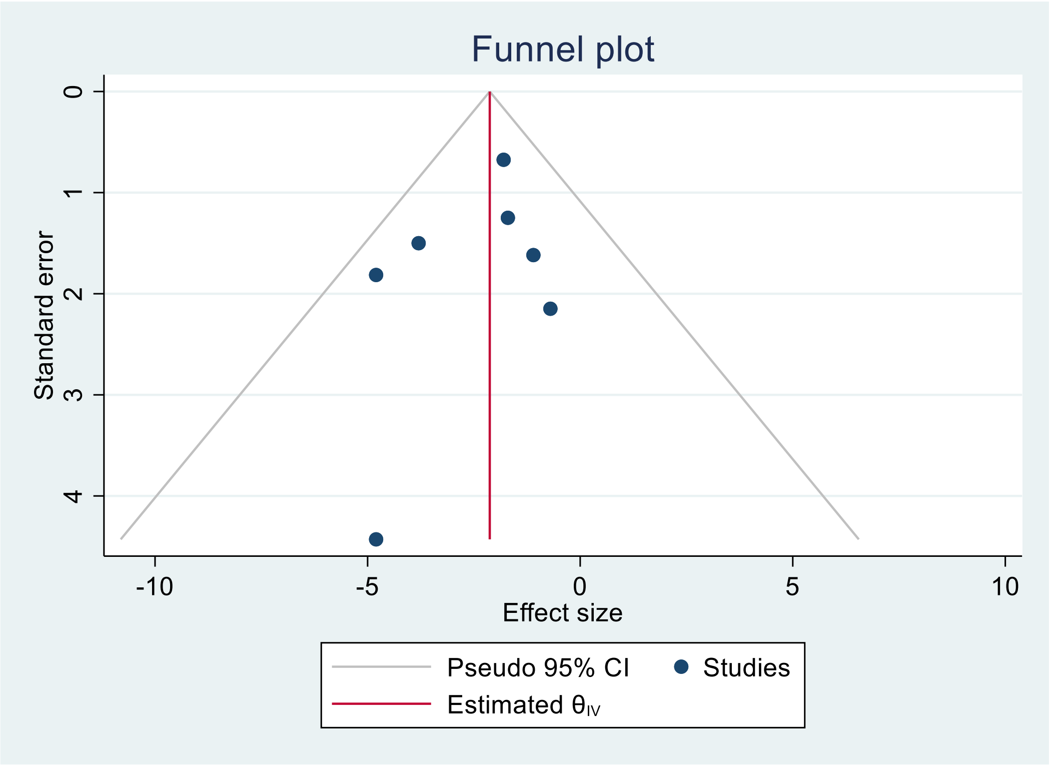

Supplement: Supplementary file 1 — Additional File Table 1: Search strategies. Additional File Table 2: Blinding Index of patients in included trials. Additional File Table 3: Metaregression according to age, male (%), diabetic patients (%), number of anti-hypertensive drugs. Additional File Figure 1: Jackknife leave-one-out sensitivity analysis. Additional File Figure 2: Subgroup analysis according to risk of bias. Additional File Figure 3: Subgroup analysis according to generation of the procedure. Additional File Figure 4: Funnel plot for primary outcomes. [file 12872_2023_3269_MOESM1_ESM.pdf]
